# Positive food challenges despite negative specific IgE testing

**DOI:** 10.1186/2045-7022-1-S1-O42

**Published:** 2011-08-12

**Authors:** Melina Makatsori, Guy Scadding, Rebecca McKenzie, Isabel Skypala, Stephen Durham

**Affiliations:** 1 Royal Brompton Hospital, Allergy, London, UK

## Background

Skin prick testing and serum food-specific IgE testing are the most commonly used diagnostic tests in evaluating IgE-mediated food reactions. However, the presence of negative tests can be falsely re-assuring - such outcomes do not always exclude allergy. Food challenges are therefore indicated. Double-blind, placebo-controlled food challenge is the gold standard for diagnosis, but in many situations, open food challenges are a more practical alternative.

## Case presentations

We present five patients, aged 27 to 58 years old, with histories of immediate-onset adverse reactions to foods. Despite having negative skin prick tests to food reagents, prick-prick tests with fresh foods and serum specific-IgE tests to the suspect foods, each individual developed symptoms, accompanied by objective signs, during open food challenges. The foods implicated included sesame, soy, chestnut, sea bass, a soft drink, and a flavoured tortilla chip. Reactions included urticaria, abdominal colic, rhinitis, and frank anaphylaxis.

The majority of patients reacted at low doses of the tested foods. Of note, all the patients except one had negative skin prick tests to common inhaled aeroallergens. The total IgE level in all cases was within the normal range (mean 46.6 iu/ml). We consider the possible reasons for the discrepancies between allergy testing and challenge testing in each case, including the lack of sensitivity of the available tests, the presence of unidentified allergens within complex, pre-prepared foods, the role of non-IgE mediated food allergy, and the possibility of psychosomatic reactions.

## Conclusion

The patient’s history remains paramount in the assessment of potential food allergy, even in the presence of negative skin prick and serum specific IgE tests. Food challenges are required to confidently exclude allergy. However, positive open challenges may produce questionable outcomes, especially in the absence of firm objective signs of an allergic response. In such cases, the double-blind, placebo-controlled challenge remains the gold-standard, despite being both labour intensive and time consuming.

